# Obstacles in the Process of Dealing With Child Sexual Abuse–Reports From Survivors Interviewed by the Independent Inquiry Into Child Sexual Abuse in Germany

**DOI:** 10.3389/fpsyg.2021.619036

**Published:** 2021-04-12

**Authors:** Wiebke Schoon, Peer Briken

**Affiliations:** Institute for Sex Research, Sexual Medicine and Forensic Psychiatry, University Medical Center Hamburg-Eppendorf (UKE), Hamburg, Germany

**Keywords:** child sexual abuse, dealing with abuse, public inquiries, Independent Inquiry into Child Sexual Abuse, qualitative research and analysis

## Abstract

Obstacles in dealing with child sexual abuse (CSA) can hinder survivors in the process of coming to terms with their experiences. The present study aims to identify and analyze factors that may pose obstacles in the long-term process of dealing with CSA. It is part of a larger research consortium “Auf-Wirkung,” funded by the German Federal Ministry of Education and Research, and was conducted in cooperation with the Independent Inquiry into Child Sexual Abuse in Germany (IICSAG). The IICSAG was appointed by the Independent Commissioner for Child Sexual Abuse Issues and the German Federal Ministry for Family Affairs, Senior Citizens, Women, and Youth in 2016. To determine responsibilities, recognize injustice, and further acknowledge the survivors of CSA in the Federal Republic of Germany (FRG) and the German Democratic Republic (GDR), the Independent Inquiry has held 1,303 private sessions with survivors of CSA by Oct. 17th, 2020. The present study focuses on exploring reoccurring problematic experiences reported by survivors in private sessions regarding the long-term process of dealing with experiences of CSA. A total of 30 transcripts of private sessions, conducted by members and appointees of the IICSAG between September 2016 and June 2019, were analyzed using qualitative content analysis. Attendants of private sessions described a variety of obstacles, including negative social reactions to disclosure, institutions' unwillingness to elucidate occurrences of CSA within their midst, as well as general financial difficulties, and those linked to redress claims. Manipulative grooming by perpetrators and limited access to adequate psychotherapy were perceived as obstructive by survivors dealing with CSA. In the context of criminal proceedings, survivors reported long durations of court proceedings and negative experiences in connection to credibility assessment. Results will be discussed to better support survivors of CSA in the process of dealing with their experiences in the future.

## Dealing With Experiences of CSA

Dealing with experiences of CSA is a continuous and lengthy process for survivors that often accompanies them throughout their lives and during which many face a variety of challenges and obstacles. Researchers have proposed a variety of empirical and theoretical models to describe and conceptualize the process of dealing with experiences of sexualized violence, with many postulating 3 or 5 different phases in which coping can occur (Horowitz, 1986; Kleber and Brom, 1992; Roth and Newman, 1993; Figley, 2013; Fischer and Riedesser, 2016). Many models see the integration of an incomprehensible experience into one's understanding of self and the world as the most meaningful part in the process of dealing with CSA. According to Figley (2013), however, “adaptation” (the last stage) can only be achieved if adequate resources (personal, social, and financial) are available. Herman (1994) agrees and claims that restoring a sense of security is of fundamental importance to survivors, including pragmatic aspects, like ensuring basic needs such as financial security. For Fischer and Riedesser (2016) the social dimension of dealing with CSA is in the foreground since the authors assume that traumatic experiences cannot be dealt with by an individual alone. Many aspects of the described models were also picked up and further developed by Gahleitner (2003). Like Fischer and Riedesser (2016) the author emphasizes the processual nature of dealing with CSA in a three-phase model. Similar to Figley (2013) she concludes that the integration of the traumatic experiences into the self-concept may only succeed with a minimum of relative security and support of at least one sustainable relationship. This conclusion is further supported by Draucker et al. (2011). Their model includes four stages (grappling with the meaning, figuring out the meaning of CSA, tackling the effects of the CSA, laying claim to one's life), five domains of functioning (life patterns, parenting, disclosure of CSA, spirituality, altruism) and six enabling factors to progress from one stage to the next. These six were contextual factors like receiving affirmative messages, having ongoing support, and experiencing a critical life event and personal factors like personal agency, personal resolves, and commitment to transcend from CSA.

In summary, giving meaning, regaining control, and being able to integrate the abuse is most relevant for survivors of sexualized violence (Horowitz, 1986; Kleber and Brom, 1992; Frazier et al., 2004; Walsh et al., 2010; Draucker et al., 2011). Conversely, the absence of enabling factors as well as any form of social and societal forms of support may hinder these relevant goals and therefore the process of dealing with CSA (Birck, 2001; Gahleitner, 2003; Draucker et al., 2011).

## Obstacles in Dealing With experiences of CSA

Obstacles in dealing with experiences of CSA are often related to factors or experiences which aggravate the symptoms and effects of the traumatic experiences, which, according to the Traumagenic Dynamics Model of Child Sexual Abuse are traumatic sexualization, betrayal, stigmatization, and powerlessness (Finkelhor and Browne, 1985). Past research was able to identify some evidence for obstacles in the process of dealing with CSA. For example, the way the social peer group or environment reacts to the disclosure of experiences of sexualized violence is important to the process of dealing with CSA. Research on the topic suggests that disclosure might be the first step in the healing process even for some individuals to regain control and to pave the way for meaning and integration of the abuse experience (Birck, 2001; Chouliara et al., 2014). Active or passive inhibition of disclosure, therefore, poses a potential obstacle in dealing with CSA. Research consistently suggests that negative reactions to disclose attempts have a lasting adverse impact on the coping process of survivors (Birck, 2001; Filipas and Ullman, 2001; Ullman et al., 2007; Ullman and Peter-Hagene, 2014). Thus, negative social reactions from a trusted or formal source are associated with greater PTSD symptoms, the relationship is mediated by maladaptive coping (Ullman and Peter-Hagene, 2014), and it reduces the likelihood that survivors will seek support (Birck, 2001). Wyatt and Mickey (1987) indicate, that a negative immediate and long-term reaction to disclosure of CSA may mediate adverse effects of the initial abuse in survivors. This may be since a person or group not believing them can be seen as a betrayal and increase the feeling of powerlessness associated with the abuse itself (Finkelhor, 1987). Consequently, negative reactions to disclosure undermine survivors' attempts to regain control and act in a self-effective way. Furthermore, social support, in general, seems important – short-term and long-term – as it may mitigate negative outcomes and lower symptomology (Murthi and Espelage, 2005).

Manipulative perpetrator behavior also called grooming, may similarly hinder the process of dealing with CSA. Wolf and Pruitt (2019) examined the effects of grooming, namely verbal coercion, grooming that used drugs/alcohol, as well as threatening/violent grooming, and found that in a linear regression model grooming categories predicted trauma symptom severity, with threatening or violent tactics having the most severe effect on survivors psychological well-being. The authors conclude that the isolation and normalization of sexually abusive interactions may aggravate the trauma and therefore hinder the healing process. These findings were supported by Chouliara et al. (2014). The authors interviewed 22 adult survivors of CSA using qualitative analysis and identified four relevant themes, amongst others “Factors hindering Recovery” and “Hurdles of Recovery.” Survivors reported that they felt insufficiently supported by family members, who oftentimes refused to address the abuse further when confronted. Furthermore, survivors feared stigmatization, especially regarding issues of mental health. Social inviolability of the perpetrator due to his or her position enabled longer abuse and prevented exposure or disclosure. Survivors reported their credibility being called into question as well as grooming and other manipulative perpetrator behavior.

Adequate psychotherapy may reduce psychological distress caused by experiences of CSA and improve the overall functioning and well-being of survivors (Price et al., 2001; Sánchez-Meca et al., 2011). Unfortunately, many survivors report a lack of access to appropriate psychological treatment options in Germany with the average waiting time for a place in a psychotherapy program being three months in larger German cities and six months in more rural areas (Pawils et al., 2017; Unabhängige Kommission zur Aufarbeitung sexuellen Kindesmissbrauchs, 2019). Additionally, Sommer (2016) illustrated the situation for individuals with complex PTSD in Germany, showing that programs and therapies often fail to successfully stabilize patients, mostly due to a lack of expertise in professionals treating survivors with these specific needs. The lack of access to adequate and efficient psychotherapy can therefore be seen as a factor hindering the process of dealing with CSA.

A factor whose influence on processing experiences of CSA has not been investigated more thoroughly are obstacles in the context of criminal investigations (Walsh et al., 2010; Görgen et al., 2012; Volbert, 2012). Some research indicates, that long-duration of court proceedings, as well as the anticipation to make a statement in front of a court (in presence of the accused perpetrator), may lead to a short-term negative influence on well-being and slower recovery of the damage caused by the crime (Runyan et al., 1988; Volbert, 2012). Furthermore, doubts about testimony or the severity of the abuse impact can be challenging for survivors, especially if there is no other “objective” evidence (Volbert, 2012). Stressful as court proceedings may be, going through with it may be associated with increased self-efficacy and restoration of control long-term (Volbert, 2012).

The present study aims to identify and analyze different obstacles in the process of dealing with CSA. It explores these reoccurring problematic experiences reported by adult survivors in the context of private sessions held by the IICSAG using openly designed interviews to encourage spontaneous statements. In this study, we focus on the analysis of contextual obstacles that survivors report in interaction with others (persons, state actors, institutions). The goal is to apply the findings to better support survivors in dealing with experiences of CSA in the future.

## Materials and Methods

### Study Design

This study was conducted in cooperation with the Independent Inquiry into Child Sexual Abuse in Germany (https://www.aufarbeitungskommission.de/english/). The Inquiry was appointed by the Independent Commissioner for Child Sexual Abuse Issues and the German Federal Ministry and consists of seven volunteer members of different subject-related fields and professions. Starting in January 2016 the Independent Inquiry called on survivors and contemporary witnesses of CSA in the GDR or FRG to contact the Inquiry staff to tell their story in form of a private session or a written report. Private sessions, with a total of 1,303 by Oct. 17th, 2020, were held in 12 different major cities by members of IICSAG and qualified representatives of the Inquiry, providing a safe space for survivors to share their experiences. Interviewers used a set of semi-structured guidelines, containing 10 topics related to the subject, including immediate and long-term consequences of CSA, disclosure, seeking support, and experiences with religious and government institutions (see Supplementary Material). The interview guidelines served primarily as a narrative-generating aid for the interviewers, intending to encourage and enable survivors to shape the pace and direction of the conversation and report as freely as possible on their experiences.

This project is funded by the German Federal Ministry of Education and Research and is part of the larger research consortium “Auf-Wirkung,” which aims to accomplish a comprehensive and extensive investigation of structural conditions in connection with sexualized violence against children and adolescents. The current study presents the first results of sub-project three, which examines recurring experiences of survivors, which may hinder the process of dealing with experiences of CSA.

### Data Selection

A set of 100 summaries of transcripts, selected by the Inquiry according to general thematic overlap, were made available to the project's research assistants. Each sub-project of “Auf-Wirkung” received 25 summaries which were then sorted according to descriptive as well as key data points and organized using excel, providing a basis for further selection. From this, 30 transcripts were chosen according to thematic overlap with the specific research questions at hand. The selected material, therefore, represents a nonprobability “convenience sampling,” meaning data was included due to availability (Robinson, 2014). Before data analysis, the research consortium “Auf-Wirkung” and each sub-project were approved by the Ethics Committee of the Department of Education at Goethe University Frankfurt.

### Data Analysis

A qualitative content analysis of 30 transcripts was conducted following Kuckartz (2018) using the qualitative data analysis software MAXQDA version 18.2.4 (www.maxqda.de/). Included private sessions were held by members of the IICSAG between September 2016 and August 2019, transcribed verbatim by a collaborating company, and made available by the Inquiry's corresponding office.

The analytic procedure of the content structuring content analysis (Kuckartz, 2018) compromises a seven-step deductive/inductive process. Firstly, the entire material was read by two researchers separately, significant text passages were marked, and thoughts were recorded in memos. Data was then structured by developing thematic main categories, based on research questions on the one hand (deductive) and on topics that emerged directly from the material (inductive) on the other (Kuckartz, 2018, p. 101–102). Using these main categories, the material was then coded sequentially by the two researchers independently, whereby multiple coding of individual text passages was possible (Kuckartz, 2018). In the next step, sub-categories were determined using inductive category development (Kuckartz, 2018, p. 72–86). For this, all text passages coded with the same main category were compiled and arranged systematically to identify recurring aspects and themes relevant to survivors in the process of dealing with CSA which had not been considered previously. Additionally, definitions for all main and sub-categories were formulated for better comprehensibility in the further course of analysis (Kuckartz, 2018). Using the resulting category system, the entire material was once again coded. After 30% of the material was coded this way, the categories were re-evaluated, revised, and definitions concretized, if necessary until the categories adequately reflected the data. The remaining material was then coded. Finally, the coded material was analyzed along with the main categories, giving an overview of all themes that emerged during the conversations regarding potential barriers in dealing with CSA (Kuckartz, 2018, p. 118). Furthermore, we explored relationships of subcategories within the main category as well as relationships between the main categories by focusing on the proximity of certain subcategories and themes within and across main categories (Kuckartz, 2018, p. 118–119). By exploring sub-categories that were often mentioned simultaneously or in proximity, we aimed to identify more complex associations between relevant themes.

## Results

### Sample and Data

The present study included 30 transcripts of private sessions held by the Independent Inquiry into Child Sexual Abuse in Germany with survivors, who report having experienced CSA in the FRG or the GDR between the 1950s and early 2000s. Table 1 displays socio-demographic and abuse context-related information. Most participants were female, 54 years on average, and half of them had children. At the time of the first abuse experience, survivors were between 7 and 13 years old on average, with one person reporting sexual abuse before age of one.

**Table 1:** Descriptive data: demographics and contexts of abuse (*N* = 30).

	***n*/*M***	**%/*SD***	**∑ = 67**
**Demographics**			
Male *(n, %)*	8	26,7	
Female *(n, %)*	22	73,3	
**Age in years**			
*M (SD)*	54,6	(11,0)	
Missing *(n,%)*	1	0,03	
**Abuse experience**			
Repeated/Multiple *(n, %)*	26	86,7	
Once *(n, %)*	4	13,3	
**Germany**			
FRG *(n, %)*	2	6,7	
GDR *(n, %)*	10	33,3	
Both *(n, %)*	1	3,3	
Missing *(n, %)*	17	56,7	
Contexts of abuse:^*^			
Family			17
Religious:			13
Catholic			8
Protestant			4
Jehovah's Witnesses			1
Federal Institutions			21
Recreational Activities			3

*n.a., not applicable; M, mean value; SD, standard*;

* *multiple entries possible*.

All 30 survivors reported long-term effects on their physical or mental health in some way or form. Pathological somatic phenomena were described most frequently including sleep disorders, pain disorders (e.g., migraine), diseases of the gastrointestinal tract, and obesity. Reports of mental health issues ranged from precise diagnoses, ascribed by survivors themselves or by clinical professionals, to implicit descriptions of general psychological discontentment. Substance abuse, anxiety, PTSD, and depression were reported frequently. Eating disorders and self-harming behavior were reported as direct or long-term consequences of the abuse. Suicidal thoughts or suicide attempts were reported by 19 out of 30 survivors and arose either while the abuse was still ongoing or as a long-term consequence within the coping process.

### Main Categories

Contextual obstacles in dealing with experiences of CSA have been organized around disclosure, how others deal with CSA, grooming, and obstacles in state legal and health care systems (see Table 2).

**Table 2:** Main- and subcategories of contextual obstacles in dealing with CSA.

**Main categories**	**Subcategories**
1. Disclosure	• Negative social reactions to disclosure • Negative consequences of disclosure • Factors enabling disclosure • Factors hindering disclosure
2. How others deal with CSA	• How the family of origin deals with CSA • How the institution deals with CSA • How society deals with CSA
3. Grooming	• Grooming the victim • Grooming the environment
4. Obstacles in state legal and mental health care system	• Obstacles in the mental health care system • Obstacles in criminal and civil law • Financial and organizational hurdles

#### Disclosure

Survivors described disclosing their experiences as a turning point in the process of dealing with CSA, either positively or negatively. Many reported a variety of immediate *negative social reactions* to their disclosure attempts, which then influenced how they continued to deal with their abuse experiences. Thus, inadequate responses to disclosure affected further disclosure attempts and therefore survivors' long-term coping process in general. Survivors especially suffered when a trusted person did not believe them.

“I then confronted her […], and asked whether she knew about it. Until the end she said that I imagined it and that I only ever caused her trouble, anyway.”

Besides immediate inadequate social reactions some survivors experienced continuing and longer-lasting negative (social) *consequences of disclosure* for example resentment and hostility from their family or social peers. Some even connected this continued distress to an aggravation of psychological symptoms.

From the reports of the participating survivors, some *factors enabling disclosure* emerged, including a change in family dynamics, seeking support from a medical or psychological professional, networking with other survivors, increasing media coverage of the topic, or the death of the perpetrator. The reports furthermore contained descriptions of *factors hindering disclosure*, including an existing difficulty or inability to put the experience into words, fear of destabilizing the family or social environment.

Or as one participant put it:

“About the family climate, I would like to tell you something briefly. In our family, the language of violence prevailed. All that was left was… silence.”

#### How Others Deal With CSA

The second theme survivors addressed was how others, more specifically the family of origin, state or church institutions, and society, deal with experiences of CSA in their midst. Survivors reported various negative ways in which *the family of origin dealt with CSA*. For example, family members ignored specific changes in the behavior of survivors, which the victims attributed to the abuse. Repressions, reinterpretations of events, or even punishments were also described. Some survivors described being blamed for the abuse by family relatives. One participant described this incident:

“And then my sister-in-law came in and saw him abusing me, […] And then […] she didn't offer help, but instead she threw him out of the apartment and accused me… I seduced him, so to speak.”

These descriptions often were connected to reports of family members trying to protect the perpetrator and his/her reputation, legacy, or career by instructing the victim not to talk about the abuse in front of other family members or friends.

“And I was instilled, ‘You don't talk about it, it's something very, very bad, something unbelievable that God doesn't want.' And you don't destroy the future for the ^*^(perpetrator) either. And you just don't tell your grandparents.'”

As seen in the quote above, survivors reported being shamed by relatives – sometimes by using god as a means to impose guilt on survivors.

Survivors reported in-depth *how institutions dealt with CSA*. From their perspective, several insufficiencies regarding actions not taken by institutions occurred. In some cases, neither any form of recognition nor adequate compensation was pursued by the institution concerned. Admissions of guilt were seldomly made. Survivors reported a general lack of transparent communication about misconduct on the part of the institution, as well as perspectives on how future protection can be secured. Moreover, survivors frequently described that they did not know whom they could have turned to for help after the experience of abuse because corresponding contact points either did not exist or were not sufficiently declared. Lastly, some survivors criticized a general lack of debate around the issue of CSA in institutions.

Survivors reported that some religious and state institutions installed structures that allowed for internal handling of allegations of abuse. It is reported how these structures were authorized by the institutions and in some catholic church institutions even replaced state or secular criminal prosecution. Within these structures, specially defined values and legal concepts prevailed and determined the respective handling of the abuse allegations, which often resulted in the protection of the perpetrator and the moral conviction of the survivors. According to survivors one goal behind this procedure was to protect the reputation of the institution at any cost.

Furthermore, survivors reported no access to appropriate sex education in childhood or adolescence and that this hindered them from disclosing their experiences. This lack of knowledge made it more difficult for survivors to recognize the abuse as an injustice at the time, which in turn reduced effort or ability to turn to other adults in search of help.

A special mention should be made about religious institutions. Some survivors denounced the taboo approach toward sexuality and sex education of catholic institutions. Survivors suspected that the taboos around sexuality contributed to a further internalization of shame and guilt regarding the experience of CSA in religious institutions. Part of this was a demonization of sexuality (outside the institutionalized framework of “marriage”), punishment systems, and corresponding induction of guilt were described.

Survivors described various barriers regarding *how society deals with CSA*. Due to a general lack of visibility of the topic within the public eye, survivors of CSA reported feeling that their experiences and interests are insignificant and irrelevant to other members of society. This lack of recognition and the feeling of being overlooked is amplified by concerns about social stigmatization.

“I always had the feeling that I had a stamp on my forehead that said: sorted out. And it took me a very long time to somehow develop a certain self-confidence where I said, ‘This is interesting… this is nobody's business'.”

Survivors described tangible situations in which they felt discriminated against and recounted prejudiced encounters as well as generally insensitive behavior toward them by employees of government service agencies. Another aspect of this experience was that survivors often had their credibility questioned – either in an official or social context, which consequently led survivors to form the impression that nobody believes them in general.

#### Grooming

Another theme that emerged during the analysis of the reports was manipulative perpetrator behavior–also known as grooming. Passages were coded only when survivors themselves described the perpetrator's behavior (subsequently) as manipulative. This included all behavior that was initiated by perpetrators to enable and facilitate abuse as well as to prevent exposure and was aimed at the child concerned or its environment. The subcategories, therefore, are “Grooming the Victim” and “Grooming the Environment,” a distinction also used by Craven et al. (2006).

In *grooming the victim* several survivors reported experiences of manipulative perpetrator behavior before, during, or in the aftermath of the abuse. Survivors described violent and non-violent types of grooming that appear to belong to a set of reoccurring strategies that perpetrators used in different phases of the abuse and with various goals.

Before the abuse, some survivors described being “selected” by a perpetrator, for example by exploiting their vulnerabilities and emotional needs.

“In retrospect, […] I think he, the ^*^(perpetrator), was always selecting, which boys were suitable for later sexual abuse. I'll say now, that he probably noticed from the start: ‘Oh, the ^*^(survivor), he is probably predestined or suitable.’”

Furthermore, perpetrators were described to deliberately form an interpersonal bond with children accompanying the abuse in some cases. Strategies described to strengthen this relationship were “payment” in form of money, gifts, or affection, treating the victims like “adults” and making alcohol, drugs, and pornographic material available. Another means used to enhance the relationship between perpetrator and survivor was the gradual isolation of the survivor from other close relatives or peers. This targeted attention led some survivors to feel a calculated increase in self-worth.

“So he kind of made sure that I was the predestined boy. And that also made me feel like I was special. So it was actually pretty… pretty ingenious, that system.”

During periods of abuse, some survivors reported being intoxicated by perpetrators to facilitate the abuse or establish compliance. Furthermore, some perpetrators normalized the abusive behavior to offer patterns of explanation that trivialized the abuse or made it seem without alternatives. Violent manipulation was also reported. Some used emotional blackmail to put pressure on survivors.

“[…] he always told me, and that was the worst thing for me, that he pressured me so much that I had to have sex with him. I understood what sex was because he loves me so much. And if I don't do that, then he will take his own life, and then my sister will be left alone with the three kids, and I can't be responsible for that and…. I think that was one of the worst things for me, that I felt responsible and then I gave myself to it.”

In the aftermath of the abuse as well as during, some survivors reported that perpetrators made them take an oath of secrecy to prevent disclosure or exposure. Besides this, the use of blackmail, threats, and other forms of violent manipulation was reported.

“He said that something would happen to me if I talked. And I believed him.”

Bribery through gifts as “rewards” were also used in some instances. There are also reports of targeted induction of shame and guilt, which gave survivors the feeling that they are partially or fully responsible for the abuse because they did not signal their unwillingness earlier. Some perpetrators insisted that survivors were the initiators of the abuse in the first place.

As well as this, the threat of punishment and consequences for the perpetrators in some cases led to the cover-up of the abuse, especially in case of partly positive emotional attachment. Additionally, perpetrators justified their behavior in religious abuse contexts by shaming victims and making them believe that perpetrators were only acting on or fulfilling “Gods will”

“And he always justified the whole thing by saying that God sees everything but can't punish everything, and that he was sort of made to do it, and that he just had to do it so that we wouldn't become evil people.”

One survivor was told that she was chosen by a higher power.

“Like, yeah, that I am chosen and will be liberated through suffering. And… through the pain I become free from sin and stuff, right? And they contribute… They contribute to that. Right.”

Apart from grooming the victim, survivors described instances of perpetrators *grooming the environment* by integrating themselves into the social environment of the survivor or deploying their societal status (e.g., chaplain, teacher, city mayor) purposefully. This sometimes led to family members protecting the perpetrator willingly or not believing the victim as illustrated in the cross-category analysis.

#### Obstacles in the State Legal System and Mental Health Care

Finally, topics that emerged from the transcripts were hurdles and obstacles regarding the state legal system and mental health care. Many survivors reported *obstacles in the mental health care system*, especially regarding psychotherapy or other counseling services like self-help groups, and the youth welfare office. Most commonly, survivors described experiencing a lack of expertise and/or sensitivity by professionals working in these areas or institutions.

Many survivors reported a lack of access to adequate psychotherapy within Germany. Furthermore, if they managed to secure a spot, some mentioned being confronted with a lack of specific qualifications of the therapist concerned, which in some cases led to a misdiagnosis or therapy that didn't fit a survivor's specific needs.

In addition, many participants described *financial and organizational hurdles*, e.g., struggles with basic income, since many survivors were unable to work due to long-term consequences of CSA and therefore needed to seek long-term financial support. In some cases, this then led to poverty, which, due to insufficient pension payments, continued into old age and meant an additional psychological burden to these survivors.

Many survivors sought financial support in the form of victim compensation, covered by the victim's compensation law (Opferentschädigungsgesetz, OEG). Almost all survivors who applied for support from the OEG reported structural problems or organizational hurdles in the application process of the procedure. Amongst other things, survivors had to undergo a variety of assessments and evaluations as part of the process. Survivors reported not being able to prove beyond a reasonable doubt that their suffering is the result of experiences of CSA or even that the abuse took place (Plausibility Check). However, this is a requirement to get support from the OEG.

Other obstacles in dealing with CSA included administrative resistance, bureaucratic hurdles, and a lack of information and support from governmental institutions and offices. Above all, this included reports on overcomplicated forms to be filled out, impenetrable bureaucratic processes, lack of networking of various official apparatus, missing information for survivors on official websites, and a lack of support in finding the appropriate information.

Survivors reported costs that were not covered by health insurance and therefore had to be paid for out of pocket. This led to high financial burdens in some cases and the discontinuation of treatment, which then, in turn, hindered survivors in their further healing process.

*Obstacles in criminal and civil law* included the duration of court proceedings, the statute of limitations, and witness credibility assessment. Survivors reported lengthy criminal proceedings, which were described as particularly exhausting and demotivating. In some cases, due to the statute of limitations of the offense(s), no report could be made, ongoing criminal prosecutions were interrupted, or affected persons refrained from filing a complaint at all.

In terms of expert evaluations and assessments, survivors reported negative experiences in assessments conducted in the process of the application for the Victim Compensation Fund (Plausibility check) as well as with regards to witness credibility assessments in criminal court cases.

Regarding the latter, survivors' criticism can be divided into two different categories: In the first, survivors reported negative experiences that they attributed to the procedure itself. In the second, those that they associated primarily with the person conducting the procedure, i.e., the assessor. Some experiences can be classified into both categories.

Aspects criticized regarding the procedure itself include the lack of clarity and consequently misunderstanding of the use of the term “null hypothesis” in credibility assessments, which describes the assumption, that the witness's report is not based on genuine experiences. This hypothesis shall then be refuted in the process. This gave survivors the impression that their testimonies were not believed from the beginning.

Furthermore, survivors reported being questioned and assessed repeatedly (police, court, witness cred.) which was experienced as extremely stressful, wearing, and led to feelings of re-traumatization in some cases.

Additionally, survivors reported gaps and discontinuities in their memory being regarded as inconsistencies during witness credibility assessment.

“The problem is credibility. […] There are always gaps in the… story, right? […] There can't be a complete picture. But that's exactly why it's not a lie, that's exactly why. You just didn't have the happiest childhood.”

Aspects criticized associated with the person conducting the procedure included lack of information regarding the procedure, insinuations of therapy-induced memories, stigmatization, and in some cases, evaluators appeared untrained or behaved insensitively.

“[…] Then she said, we have to conduct an expert evaluation. And I didn't even realize at that moment that this was going to be a credibility assessment, right?”

Additionally, negative experiences during the credibility assessment in some cases led to a general skepticism toward various official procedures, which then affected other areas in the process of dealing with CSA.

An important part was also that survivors felt they did not have any control over the process:

“And then, though, that loss of control, that's just that again, that loss of control, knowing exactly, I can't do anything, I'm standing there again, I can't do anything. I have to surrender to it.”

Overall obstacles in the state legal system and mental health care were reported to negatively impact the process of dealing with experiences of CSA for survivors.

### Cross-Category Analysis

In addition to analyzing the main themes, we also examined the relationships between the different categories to provide a more contextualized and comprehensive picture of experiences and factors posing as contextual obstacles in the process of dealing with experiences of CSA. Doing this it became clear that most of the categories were interconnected and interacted in certain ways. For example, *how the family dealt with CSA* was highly related to *disclosure* and specifically, *factors hindering disclosure* and *consequences of disclosure*. Thus, many of the described negative behaviors by family members happened in the context of disclosure attempts, for example, the instructions to either never talk about the abuse or stop talking about it in the future.

“Then at some point she said, ‘Stop stirring up old stories.' And, ‘You need to lay these stories to rest now…'”

There is also an overlap between *how the family of origin deals with CSA* and *grooming the environment* since survivors on some occasions reported family members being close to and friendly with the perpetrator or perpetrators blatantly and successfully discrediting the survivors' credibility in front of family members.

“And I wanted to clarify things, or at least understand them, and I caused a lot of turmoil in the family. And I was often told to stop now and […] that I was the crazy one and that I should go to my therapy and finally stop digging around in the old story.”

This also occurred in connection with *how institutions deal with CSA*. Through a high social status or institutionalized authority and the social prestige that goes along with it, perpetrators were in some cases protected from any consequences. Oftentimes perpetrators were more likely to be believed than the survivor and consequently acquitted of any guilt by the institution. Furthermore, to protect the reputation of the institution, criminal prosecution was sometimes waived.

*Grooming the victim* was also related to *factors hindering disclosure*. By inducing shame and guilt, using violent threats, or acquiring an oath of secrecy from survivors, perpetrators tried to prevent disclosure and exposure in some cases. The internalization of feelings of shame and complicity in some cases created an illusion of consensuality that perpetrators exploited. In summary, factors, and experiences that may hinder the process of dealing with CSA are located on many levels and interact with each other in a complex way.

## Discussion

Using qualitative methods, the present study aimed to identify and analyze factors and experiences that may hinder the process of dealing with experiences of CSA. Four main themes which had 12 subthemes were analyzed in this study. Our analysis showed that the categories are mostly interconnected and interact with each other on various levels. Survivors of CSA reported being confronted with a variety of obstacles, throughout the process of dealing with their experiences with some obstacles occurring simultaneously, repeatedly or in a cumulative way.

To better understand how the experiences described by self-identified survivors may negatively influence the process of dealing with CSA it is useful to take a closer look at the Traumagenic Dynamics Model of CSA by Finkelhor and Browne (1985) as well as the CSA healing model by Draucker et al. (2011). The first provides a particularly suitable framework as to why the described experiences are perceived as problematic by survivors, while the second might explain how the identified factors may hinder the process of dealing with CSA.

Our results suggest that disclosure, or rather negative social reactions to disclosure attempts, pose an obstacle in dealing with CSA. This is mostly because survivors get discouraged to share their story with anyone else out of shame, after being confronted with resentment and disbelief by loved ones or formal sources (Birck, 2001). This seems to be because the internalized stigma and the utmost feeling of betrayal connected to the traumatic experience are relived in moments of these unsuccessful disclosure attempts (Finkelhor and Browne, 1985). In their CSA healing model Draucker et al. (2011) postulate, that receiving at least one “affirmative message” from a trusted person or formal source can function as an important enabling factor and therefore help survivors to progress in the process of dealing with their experiences. These findings are supported by Ullman and Peter-Hagene (2014), who found that negative social reactions to disclosure of abuse were related to greater PTSD symptoms, with survivors perceiving less control over their dealing process. Furthermore, Sivagurunathan et al. (2019) identified feelings of shame, guilt, and self-blame, a higher social standing of the perpetrator as well as negative social reactions as obstacles affecting disclosure in male survivors of CSA. Our findings are also supported by Chouliara et al. (2014) who recognized disclosure as a major factor in enabling the healing process for survivors of CSA with negative social reactions posing as an obstacle. Apart from being confronted with continuing resentment and disbelief, survivors reported being shamed or humiliated by family relatives, who sometimes supported the offender rather than the survivor. This implies that survivors also relived stigmatization, betrayal, and also powerlessness in these situations (Finkelhor and Browne, 1985). Another enabling factor identified by Draucker et al. (2011) is “ongoing support,” which according to the authors goes beyond a one-time affirmative message but describes the feeling of trusted persons being there for the survivor regardless. Our results are supported by a study by Schönbucher et al. (2014) which indicates that adolescents who experienced CSA wish for more support from their parents.

Our data further indicate that state and religious institutions frequently ignored survivors and didn't support them sufficiently. They seldomly took adequate action to elucidate cases of abuse in their midst or compensate survivors, which made survivors feel powerless and betrayed (Finkelhor and Browne, 1985). This is most likely connected to the power imbalance that often prevailed and still in some cases prevails in some institutions, summarized under the term clericalism by Dreßing et al. (2018) for catholic church institutions. This describes a hierarchical-authoritarian system that enabled priests in superior positions to dominate unconsecrated persons and promoted secrecy, cover-ups, and unsuitable reactions. Comparable structures and their connection to sexual abuse, as well as the use of additional repressive measures to ensure the silence of survivors, can also be found in children's homes and other state institutions in the FRG and GDR (Wazlawik et al., 2014; Hackenschmied et al., 2018; Sachse et al., 2018). Since many of the events reported by survivors took place a long time ago, it can be assumed that the circumstances in some institutions have changed considerably, while other institutions (e.g., those in the GDR) no longer exist. Nevertheless, Nagel et al. (2021) point out, that important insights can be drawn from reports of survivors which should be taken into account in the development of institutional prevention programs today.

Manipulative perpetrator behavior may also pose as an obstacle in the process of dealing with CSA, mainly by delaying or hindering disclosure. Our findings implicate that perpetrators used a variety of violent and non-violent strategies to facilitate abuse and prevent exposure. Besides, perpetrators induced shame and guilt by creating an illusion of consensuality, instilling in victims the feeling of being actively and willingly involved in the abuse. This shame and guilt oftentimes kept survivors from disclosing and regaining self-worth, self-efficacy, and restoration of self-confidence, thus aggravating the feeling of powerlessness and stigma (Finkelhor and Browne, 1985). These findings are supported by Schröder et al. (2020b) who found that perpetrators used similar strategies to prevent exposure in the context of organized and ritual CSA. In addition, Wolf and Pruitt (2019) found that especially violent grooming in form of threats predicts higher trauma symptoms, anxiety, depression, sleep problems, and dissociative issues in survivors of CSA. Draucker et al. (2011) describe “personal agency” as another factor enabling survivors to eliminate shame and guilt by acknowledging that what happened to them was wrong and that they are not at fault. A feeling of personal agency therefore could help survivors to regain controllability and to fight the feeling of powerlessness (Finkelhor and Browne, 1985; Frazier et al., 2004; Draucker et al., 2009).

Lastly, survivors reported several obstacles regarding the state legal system and mental health care in Germany. The most pressing issues being a lack of access to adequate (psycho)therapy, financial burdens, duration of court proceedings, and witness credibility assessment as well as plausibility checks. These findings are consistent with research by Görgen et al. (2012) who showed, that apart from the better documented psychological and physical long-term effects of CSA financial burdens and other long-term economic consequences may play a substantial role in the process of dealing with CSA but haven't been examined in detail yet. In line with this Herman (1994) as well as Figley (2013) stated that financial, just as much as social and personal resources, are necessary for survivors to rebuild a safe environment for themselves and help them integrate their experiences in self and world. Our data illustrate how financial burdens acted as a direct obstacle, as they prevented survivors from regaining their sense of security (Herman, 1994; Gahleitner, 2003; Figley, 2013), and as an indirect obstacle, as they e.g., hindered survivors from accessing suitable psychotherapy.

Despite several measures taken to better support survivors in recent years, negative experiences in court proceedings were reported by survivors. This finding is supported by the results of a study by Dreßing et al. (2018) in which survivors from a wider age range also reported experiencing stress and discomfort as well as long-term dissatisfaction with the court proceedings and judicial outcome in some cases. This may be due to measures not being implemented properly in practice, measures not being communicated by victim representatives, or victim-oriented discussion awakening expectations in survivors that cannot be fulfilled (Volbert, 2012). The author concludes that many obstacles and hurdles often reported by survivors of CSA describe factors that are inherent to the legal process and therefore cannot be eradicated easily.

Nevertheless, it seems important to point out, that not being able to control the legal process may enhance the feeling of powerlessness in survivors (Finkelhor and Browne, 1985). Regaining control as part of personal agency therefore might also play an important role in assessment or evaluation situations (witness credibility assessments, plausibility check) (Draucker et al., 2011). Survivors frequently described these experiences as reinforcing the belief that they are not believed, accompanied by a deep-rooting feeling of loss of control. They feared being unable to influence the alleged “outcome” of the process, which in combination with missing information about the procedure and partial insensitivity by experts led to severe stress for these survivors (see also Schröder et al., 2020a). Furthermore, the procedure of witness credibility assessment in Germany has been criticized over the years, some authors suggest evaluating the procedure for a diverse group of survivors and specific circumstances (Schoon and Briken, 2019).

Our cross-category analysis showed that many of the identified factors interact with each other in a layered and complex way. Most notably many themes interact with factors hindering disclosure. This result is supported by Alaggia et al. (2019) who analyzed 33 studies between 2000 and 2016 examining factors influencing disclosure of CSA and found that amongst other things a close, family-like relationship to the perpetrator, shame, self-blame, fear of negative consequences, and stigma, dysfunctional family communication and a general lack of discussion about sexuality within society are obstacles for disclosing experiences of CSA at any given point. Similar results have been shown by Birck (2001) who interviewed 22 women who had experienced “sexualized violence in the context of a relationship of trust” in their childhood and who had completed psychotherapy as adults. She could show that disclosure was often followed by denial responses (disbelief, defensiveness, blaming the victim) by the family of origin especially. Nevertheless, disclosing their experiences was perceived as liberating and empowering by survivors, especially in the long-term perspective. Birck (2001) concludes, that disclosure is an important milestone in the process of dealing with CSA. Especially since the reactions to disclosure, positive or negative, influence the further process in a significant way. Thus, those who received an affirmative message were more likely to seek therapeutic help while those who experienced negative reactions often decided never to talk about it again. This matches the enabling factors “affirmative message” and “ongoing support” identified by Draucker et al. (2011).

All obstacles identified in this study contain aspects of the traumatic experience (traumatic sexualization, stigmatization, powerlessness, and betrayal) and thus a reliving of it (Finkelhor and Browne, 1985). They may thus prevent survivors from integrating the traumatic experiences into their self-concept and moving forward in the process of dealing with experiences of CSA (Gahleitner, 2003; Draucker et al., 2011). All this could be an explanation for why survivors of CSA often report that the experiences following the abuse were at least as bad as the abuse itself (Birck, 2001).

## Limitations

The material used in this study represents a cross-sectional sample and was not primarily collected for scientific purposes. This poses a methodological challenge. Nevertheless, it enables an impartial approach to experiences of sexualized violence from the perspective of survivors in a unique way.

Additionally, the current sample “suffers” from selection bias on different levels. Firstly, survivors who responded to the Commission's call to tell their story to the Independent Inquiry in a private session might differ from those who did not. Whether a survivor decided to share their experiences using one of the available formats might be linked to numerous and heterogeneous motives we can neither identify nor anticipate in hindsight.

Furthermore, the sample might be prone to gender bias as well. Up to this point, 83% of the survivors who shared their story with the IICSAG were female and 16% male. However, this is not representative. We have twice as many female survivors as male survivors in our sample. We did not address potential gender differences regarding obstacles. Additionally, participants in our sample were 54 years old on average. This means that in most cases experiences of CSA go back up to 45 years and therefore relate to structures and circumstances that no longer exist today. Nevertheless, our results implicate that many obstacles and hurdles survivors reported still exist today and are encountered by survivors of diverse contexts in a reoccurring pattern in the process of dealing with experiences of CSA.

Besides, the study included only a small sample out of 1,303 private sessions due to the scope of the framework of the research consortium “Auf-Wirkung.” Furthermore, the sample was not picked randomly, since only a fraction of the private sessions has been transcribed verbatim. The data therefore cannot be regarded as representative and generalized conclusions are not possible. Furthermore, due to the sensitivity of the subject, we were able to get permission to quote only from a few participants.

Among other reasons the open question format and the unstructured interview style, have ensured that only a superficial exploration of what survivors may themselves interpret as hindering has taken place. On the other hand, the detailed description of a variety of obstacles implies a high relevance of these issues for the survivors interviewed in this setting.

## Practical Implications

Our findings suggest that creating safe spaces and opportunities for disclosure could be helpful in more than one way for survivors in the process of dealing with experiences of CSA (Chouliara et al., 2014). In the case of state and religious institutions this could be achieved for example by these institutions ensuring that, as part of prevention programs, suitable contact persons are available for potential victims of sexualized violence. Furthermore, given the replicated reports of lack of access to therapy a potential expansion of the availability of suitable therapy places for survivors of CSA in Germany could be discussed (Görgen et al., 2012). Focusing on low-threshold offers and specific expertise could be helpful (Pawils et al., 2017; Alaggia et al., 2019). Insights and research regarding obstacles in the process of dealing with CSA and their meaning for survivors could be part of targeted training for clinicians. Institutions willing to process cases of child sexual abuse in the past are recommended to ensure the participation of survivors as a part of recognition (Kavemann et al., 2019). It should be noted that in recent years there have been several studies examining specific structures enabling abuse in individual institutions also formulating recommendations for the further handling of reprocessing (Keupp et al., 2017; Dreßing et al., 2018; Rau et al., 2019). Our results suggest that it could further be useful to provide information about grooming strategies and their long-term effects on survivors as part of prevention and rehabilitation processes as well as include these in training programs of clinicians. Service providers and governmental agencies also play a role in supporting and guiding survivors in the process of dealing with experiences of CSA. Therefore we agree with Sivagurunathan et al. (2019) and believe it useful to educate personnel accordingly. Concerning witness credibility assessment, our findings suggest that educating evaluators about the aspects of the evaluation process survivors describe as obstacles and needs of survivors could be helpful. Potentially, a uniform certification of credibility assessors in Germany could also be discussed after further researching this specific topic.

## Conclusion

Our findings are consistent with prior research on the topic and first and foremost support the assumption that survivors of CSA in Germany encounter a variety of obstacles in the process of dealing with their experiences. These obstacles may actively or passively hinder survivors to integrate their experiences in their concept of self and world and reestablish controllability over their lives. Regaining control is a counterpart to experiences and feelings of persistent powerlessness and therefore significant in the recovery process of survivors of CSA. While some of the findings can be supported by existing research, others have not been investigated thoroughly, yet. Among these are financial obstacles, lack of access to adequate psychotherapy, aspects regarding the (German) legal state system (e.g., duration of judicial court proceedings, witness credibility assessment). All the factors and recurring experiences identified in this study can be viewed individually, but their influence on the process of dealing with CSA becomes clear when they are considered in their complexity. Future research may focus on survivors with a wide range of backgrounds in contexts, using a structured interview potentially asking specifically about obstacles in dealing with experiences of CSA.

## Data Availability Statement

The dataset consists of transcripts with survivors of child sexual abuse. Only the project researchers are allowed to view and analyze the data. The Data set can not be made available to anyone outside the research team. Questions regarding the datasets should be directed to w.schoon@uke.de.

## Ethics Statement

The studies involving human participants were reviewed and approved by Ethics Committee of the Department of Education at Goethe University Frankfurt. The patients/participants provided their written informed consent to participate in this study.

## Author Contributions

PB designed the study. Data was collected by PB and the IICSAG. WS and PB analyzed and interpreted the data. WS wrote the initial draft of the manuscript in constant consultation with PB. PB and WS had full access to all the data in the study and take responsibility for the integrity of the data and the accuracy of data analysis. All authors have contributed to, read, and approved the final version of the manuscript.

## Conflict of Interest

PB is a member of the IICSAG. The remaining author declares that the research was conducted in the absence of any commercial or financial relationships that could be construed as a potential conflict of interest.

## Abbreviations

IICSAG: Independent Inquiry into Child Sexual Abuse in Germany
CSA: Child Sexual Abuse
FRG: Federal German Republic
GDR: German Democratic Republic
OEG: Opferentschädigungsgesetz (victims’ compensation law).
