# Supplementary material for: Obstacles in the Process of Dealing With Child Sexual Abuse–Reports From Survivors Interviewed by the Independent Inquiry Into Child Sexual Abuse in Germany
Source: Front Psychol. 2021 Apr 12;12:619036. doi: 10.3389/fpsyg.2021.619036 (PMC8072217; doi:10.3389/fpsyg.2021.619036)
Supplement: Supplementary file 1 [file Data_Sheet_1.PDF]

UNABHÄNGIGE KOMMISSION
ZUR AUFARBEITUNG
SEXUELLEN KINDESMISSBRAUCHS

Interview Guidelines for Private Sessions

Survivors and contemporary witnesses of sexual abuse can speak at a confidential hearing without being obliged to follow any instructions. You decide for yourself what you would like to report.

However, it can be helpful if there is some orientation beforehand. The following guiding questions can serve this purpose:

1. What led you to register for the hearing and report the sexual abuse you experienced?
2. At this point, if you wish, you can talk about the assault you experienced. In doing so, you do not have to give any details about the acts.
3. How did the abuse stop?
4. To whom and when did you first disclose the sexual abuse?
5. What were the reactions when you talked about the abuse?
6. Did you seek help and support, and did you get it?
7. What was your experience with relevant authorities and support institutions?
8. What consequences does the abuse have or did it have for you?
9. Does the sexual abuse still play a role in your life today?

For further information visit:

www.aufarbeitungskommission.de/english/
