# Supplementary material for: Obstacles in the Process of Dealing With Child Sexual Abuse–Reports From Survivors Interviewed by the Independent Inquiry Into Child Sexual Abuse in Germany
Source: Front Psychol. 2021 Apr 12;12:619036. doi: 10.3389/fpsyg.2021.619036 (PMC8072217; doi:10.3389/fpsyg.2021.619036)
Supplement: Supplementary file 2 [file Data_Sheet_2.PDF]

UNABHÄNGIGE KOMMISSION
ZUR AUFARBEITUNG
SEXUELLEN KINDESMISSBRAUCHS

GESCHICHTEN
DIE ZÄHLEN

VERTRAULICHE ANHÖRUNG
LEITFRAGEN

Betroffene sowie Zeitzeuginnen und Zeitzeugen können im Rahmen einer vertraulichen Anhörung ohne Vorgaben sprechen. Was Sie berichten möchten, entscheiden Sie selbst.

Es kann jedoch hilfreich sein, wenn es vorher eine gewisse Orientierung gibt. Dazu können die folgenden **Leitfragen** dienen:

1. Was hat Sie bewogen, sich zur Anhörung anzumelden und über den sexuellen Missbrauch, den Sie erlebt haben, zu berichten?

2. Wenn Sie möchten, können Sie an dieser Stelle von den erlebten Übergriffen erzählen. Dabei müssen Sie keine Angaben zu den Taten machen.

3. Wie kam es dazu, dass der Missbrauch aufgehört hat?

4. Wem und wann haben Sie zum ersten Mal über den sexuellen Missbrauch berichtet?

5. Wie waren die Reaktionen, als Sie über den Missbrauch gesprochen haben?

6. Haben Sie Hilfe und Unterstützung gesucht und haben Sie sie bekommen?

7. Welche Erfahrungen haben Sie mit zuständigen Behörden und Unterstützungseinrichtungen gemacht?

8. Welche Folgen hat oder hatte der Missbrauch für Sie?

9. Spielt der sexuelle Missbrauch heute noch eine Rolle in Ihrem Leben?

GESCHICHTEN
DIE ZÄHLEN

Weitere Informationen:

www.aufarbeitungskommission.de

Infotelefon Aufarbeitung:

0800 4030040 (anonym und kostenfrei)
